# Synthesis of CdSe Quantum Dots Using *Fusarium oxysporum*

**DOI:** 10.3390/ma9100855

**Published:** 2016-10-20

**Authors:** Takaaki Yamaguchi, Yoshijiro Tsuruda, Tomohiro Furukawa, Lumi Negishi, Yuki Imura, Shohei Sakuda, Etsuro Yoshimura, Michio Suzuki

**Affiliations:** 1 Department of Applied Biological Chemistry, Graduate School of Agricultural and Life Sciences, The University of Tokyo, 1-1-1 Yayoi, Bunkyo-ku, Tokyo 113-8657, Japan; kenmei.takaaki@gmail.com (T.Y.); 2.obv.0627@gmail.com (Y.T.); mountainXTC@live.jp (T.F.); aimuray@mail.ecc.u-tokyo.ac.jp (Y.I.); asakuda@mail.ecc.u-tokyo.ac.jp (S.S.); ayoshim@mail.ecc.u-tokyo.ac.jp (E.Y.); 2 Institute of Molecular and Cellular Biosciences, the University of Tokyo, 1-1-1 Yayoi, Bunkyo-ku, Tokyo 113-0032, Japan; lnegishi@iam.u-tokyo.ac.jp; 3 Faculty of Liberal Arts, the Open University of Japan, 2-11 Wakaba, Mishima-ku, Chiba-city, Chiba 261-8586, Japan

**Keywords:** CdSe, *Fusarium oxysporum*, quantum dot, superoxide dismutase

## Abstract

CdSe quantum dots are often used in industry as fluorescent materials. In this study, CdSe quantum dots were synthesized using *Fusarium oxysporum*. The cadmium and selenium concentration, pH, and temperature for the culture of *F. oxysporum* (*Fusarium oxysporum*) were optimized for the synthesis, and the CdSe quantum dots obtained from the mycelial cells of *F. oxysporum* were observed by transmission electron microscopy. Ultra-thin sections of *F. oxysporum* showed that the CdSe quantum dots were precipitated in the intracellular space, indicating that cadmium and selenium ions were incorporated into the cell and that the quantum dots were synthesized with intracellular metabolites. To reveal differences in *F. oxysporum* metabolism, cell extracts of *F. oxysporum*, before and after CdSe synthesis, were compared using sodium dodecyl sulfate-polyacrylamide gel electrophoresis (SDS-PAGE). The results suggested that the amount of superoxide dismutase (SOD) decreased after CdSe synthesis. Fluorescence microscopy revealed that cytoplasmic superoxide increased significantly after CdSe synthesis. The accumulation of superoxide may increase the expression of various metabolites that play a role in reducing Se^4+^ to Se^2−^ and inhibit the aggregation of CdSe to make nanoparticles.

## 1. Introduction

CdSe quantum dots—semiconductor nanoparticles with special optical properties of size-dependent luminescence [1,2]—are used for biological imaging, solar cells, electronics, and photonics [3,4,5,6,7,8]. They are synthesized using chemical methods at present, but there are some problems such as production of toxic byproducts and the requirement of high temperature and pressure for the synthesis [9,10,11]. Therefore, more environmentally friendly methods to obtain CdSe quantum dots are desired.

Recently, many researchers have reported that various microorganisms can produce CdSe quantum dots. These methods using microorganisms are performed at ambient temperature and pressure and do not require toxic solvent. In addition to these merits, these CdSe quantum dots are likely to interact with other biomacromolecules, such as enzymes and antibodies, because synthesis by microorganisms results in a number of attached organic substances derived from the living cells [12,13].

Kumar et al. (2007) reported the synthesis of highly luminescent CdSe quantum dots at room temperature by *Fusarium oxysporum* with a mixture of CdCl_2_ and SeCl_4_ [14]. Cui et al. (2009) used living yeast cells as a biosynthesizer of CdSe quantum dots at 30 °C [15]. Pearce et al. (2008) reported that the anaerobic bacterium, *Veillonella atypica*, reduced selenium oxyanions to form nanospheres of selenium. These nanospheres were further reduced by the bacterium to form reactive selenide, which could be precipitated with a suitable metal cation, such as Zn^2+^, to form zinc selenide with optical and semiconducting properties [16]. Ayano et al. (2014 and 2015) isolated the bacterium, *Pseudomonas* sp. RB, most responsible for synthesizing CdSe from a culture medium containing selenite and cadmium [17,18]. These environmentally friendly biosynthetic methods do not use combustible, explosive, or toxic organic reagents at high temperature and pressure.

However, the methods using microorganisms can have a higher cost, because the culture of microorganisms uses an appreciable amount of a sterilized culture medium. Thus, it is necessary to optimize culture conditions and add functional value to CdSe quantum dots. To improve the microorganism-dependent synthetic production of CdSe quantum dots, it will be important to understand their generation on a molecular level. These researches will be useful to decrease the cost and to create novel functional CdSe quantum dots. In this study, we optimized the synthetic conditions for the generation of CdSe quantum dots using *F. oxysporum* (*Fusarium oxysporum*) and analyzed the proteomic profile of *F. oxysporum* before and after synthesis of these quantum dots.

## 2. Materials and Methods

### 2.1. Culture of F. oxysporum (Fusarium oxysporum)

*F. oxysporum* (JCM11502) was used for the synthesis of CdSe quantum dots. The mycelium of *F. oxysporum* was cultured on the oatmeal plate (3% oatmeal/1.5% agar). To prepare the spores, the mycelium grown on the oatmeal plate was transferred to carboxymethylcellulose (CMC) liquid medium (1.5% CMC/0.1% yeast extract/0.1% NH_4_NO_3_/0.1% KH_2_PO_4_/0.05% MgSO_4_), which was cultivated with continuous shaking (300 rpm) at 26.5 °C for 72 h. The culture broth was filtered by miracloth (Calbiochem, Darmstadt, Germany) and obtained spores were suspended in a 30% glycerol solution at the concentration of 2000 spores/µL. An amount of 1 mL of the spore solution was added to 3 mL of oatmeal liquid medium and incubated with continuous shaking (140 rpm) at 25 °C for 24 h for pre-culture. After 24 h, the pre-culture broth (3 mL) was transferred into 250 mL of an oatmeal liquid medium and incubated with continuous shaking (140 rpm) at 25 °C for 48 h for main-culture.

### 2.2. Synthesis of CdSe Using F. oxysporum

The mycelia of *F. oxysporum* after main-culture were collected by filtration with miracloth and washed with distilled water to remove the liquid medium. The washed mycelia (20 mg (wet weight)/L) were suspended in various concentrations of cadmium ion (CdCl_2_) and/or selenium ion (Na_2_SeO_3_ or Na_2_SeO_4_) solution. The mixture was incubated on the shaker (140 rpm) for 24 h. The pH of each solution was controlled by buffers. The buffer of pH 4.0 was adjusted by a 50 mM potassium hydrogen phthalate solution. The buffer of pH 6.0 was adjusted by a 50 mM sodium phosphate solution. The pH 7.5 was adjusted by a 50 mM 4-(2-hydroxyethyl)-1-piperazineethanesulfonic acid (HEPES) solution. The pH 9.0 was adjusted by a 50 mM Ches solution.

### 2.3. Detection of Fluorescence from F. oxysporum

The mycelial cells of *F. oxysporum* after the treatment of cadmium ion and/or selenium ion solution were observed under the ultra violet (UV) light (wavelength: 365 nm, Transilluminator, 2UV). After the observation of fluorescence, the fungus cells of *F. oxysporum* were broken with ultrasonication (U200S control, IKA LABOTECH INK, Staufenberg, Germany). After centrifugation to remove the precipitation, fluorescence emission spectra of the supernatant were measured from 350 to 500 nm at an excitation wavelength of 310 nm using a fluorescence spectroscopy photometer (spectrophotometer FP-6500, JASCO, Tokyo, Japan).

### 2.4. Observation of Transmission Electron Microscopy (TEM)

Transmission Electron Microscopy (TEM) observations were performed using a JEOL JEM-2010 TEM (JEOL, Tokyo, Japan) operated at 200 kV. The images were recorded in a charge coupled device (CCD) camera (Gatan ESW-500 W, Gatan, Sarasota, FL, USA) as digitized images. An X-ray chemical analysis in the TEM was performed using an ultra-thin window type energy-dispersive spectrometer (EDS) (JEOL JED 2200) equipped to the TEM.

### 2.5. Preparation of Ultrathin Cross Section of F. oxysporum

Mycelia of *F. oxysporum* treated with or without cadmium and selenium ions solution were fixed in a 50 mM HEPES buffer (pH 7.5) containing 4% glutaraldehyde at room temperature for 1 h and were then washed three times in a 50 mM HEPES buffer (pH 7.5) for 10 min. The fixed mycelia were incubated in 50%, 70%, 80%, 90%, and 100% ethanol for 10 min. The dehydrated mycelia were incubated in propylene oxide for 10 min, propylene oxide:epoxy resin (3:1) for 3 h, propylene oxide:epoxy resin (1:1) for 3 h, propylene oxide:epoxy resin (1:3) for 3 h. Finally, the samples were embedded in epoxy resin and heated at 70 °C for 3 days. Ultrathin sections were prepared by using an ultramicrotome fitted with a glass knife.

### 2.6. Purification of CdSe Quantum Dots Using Gel-Filtration Chromatography

The extracts from *F. oxysporum* after ultrasonication were subjected to gel-filtration high performance liquid chromatography (HPLC) on a TSKgel G2000SWXL (7.8 mm × 300 mm, TOSOH, Tokyo, Japan). Elution was performed with a 50 mM ammonium acetate buffer solution (pH 5.7) at a flow rate of 0.6 mL/min and monitored by measuring fluorescence at excitation 310 nm/emission 410 nm.

### 2.7. Separation of Proteins from F. oxysporum Using Sodium Dodecyl Sulfate-Polyacrylamide Gel Electrophoresis (SDS-PAGE)

Mycelia of *F. oxysporum* treated with or without cadmium and selenium ions were resuspended in 5 mL of 1.5 M aqueous NaCl solution containing 100 mg of yatalase (TaKaRa Bio, Shiga, Japan) and 100 mg of lysing enzymes from *Trichoderma harzianum* and incubated on a tube rotator at 28 °C for 3 h. After centrifugation, the obtained precipitates containing protoplasts were suspended in 500 µL of a Tris/NaCl/ethylenediaminetetraacetic acid (EDTA) (TNE) buffer (1% NP-40, 20 mM Tris-HCl (pH 7.4), 1 mM EDTA, 150 mM NaCl and 1% protease inhibitor cocktail) and were broken by ultrasonication. After centrifugation, the supernatant was applied to SDS-PAGE.

### 2.8. Identification of the Protein Using Liquid Chromatography/Mass/Mass (LC/MS/MS) Analysis

The protein band on SDS-PAGE described above was excised from the gel, destained according to the protocol by the SilverQuest Silver Staining Kit (Invitrogen, Carlsbad, CA, USA), dehydrated with acetonitrile, and dried. The residue was treated with 50 µL of a reducing solution (10 mM dithiothreitol (DTT) and 100 mM NH_4_HCO_3_) for 1 h at 56 °C and 50 µL of an alkylating solution (55 mM iodoacetamide and 100 mM NH_4_HCO_3_) for 45 min at room temperature in the dark. The residue was further treated successively with 100 mM NH_4_HCO_3_ (100 µL) for 10 min, acetonitrile (100 µL) for 15 min, 100 mM NH_4_HCO_3_ (100 µL) for 10 min, and acetonitrile (100 µL) for 15 min and dried. Proteins in the gel were digested overnight at 37 °C with 10 µL of 10 µg/mL Trypsin Gold (Promega, Madison, WI, USA) in 50 mM NH_4_HCO_3_. The liquid portion of the reaction mixture was transferred to a new tube. The remaining gel residue was extracted three times with 20 µL of a water solution containing 50% acetonitrile and 0.05% trifluoroacetic acid (TFA). The latter extracted solution was pooled with the former liquid portion and dried. Residual pellet was dissolved in 20 µL of a water solution containing 2% acetonitrile and 0.05% TFA, and was vortexed for 5 min. After centrifugation, the supernatant was transferred into an analytical vial, and a liquid chromatography/mass/mass LC/MS/MS analysis was performed in an Orbitrap VEROS ETD system (Thermo Fisher Scientific, Carlsbad, CA, USA).

Proteome Discoverer 1.4 (Thermo Fisher Scientific) was used for protein identification. *F. oxysporum* protein data from the web site of EnsembleFungi (http://fungi.ensembl.org/Fusarium_oxysporum/Info/Index) were used as protein databases. 

### 2.9. Visualization of Superoxide in Mycelia of F. oxysporum

An amount of 5 µM MitoSOX (Molecular Probes, Carlsbad, CA, USA) in Hanks’ Balanced Salt Solution (HBSS, Gibco, Carlsbad, CA, USA) was prepared according to the manufacturer’s instructions and used for detection. An amount of 30 mM dihydroethidium (DHE) dissolved in anhydrous dimethyl sulfoxide (DMSO) was diluted 1000-fold with HBSS and used for detection. Mycelia of *F. oxysporum* treated with or without cadmium and selenium ions were harvested by filtration, washed with water, and incubated with HBSS containing 5 µM mitoSOX or 30 µM DHE for 10 min at 37 °C in the dark. Mycelia were washed with HBSS and applied to microscope slides. To obtain images, an IX71 (Olympus, Tokyo, Japan) fluorescence microscope equipped with a DP70 camera (Olympus) was used. The excitation filter (BP360-370, Olympus) and emission filter (BA420, Olympus) were used for detection of fluorescence from both mitoSOX and DHE. The camera exposure time was set at 1 s for both mitoSOX and DHE fluorescence.

## 3. Results and Discussion

**Figure 1:**
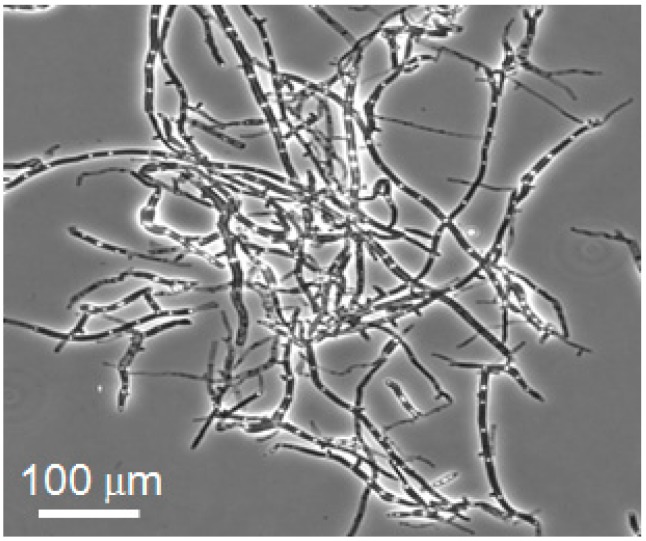
The mycelial cells of *F. oxysporum* (*Fusarium oxysporum*) observed by an optical microscope.

*F. oxysporum* was cultured to obtain mycelial cells (Figure 1), and the mycelial cells were washed with distilled water and incubated in a solution containing cadmium and selenium ions under various conditions. The fluorescence of mycelial cells was verified with a transilluminator and fluorescence spectrophotometer.

**Figure 2:**
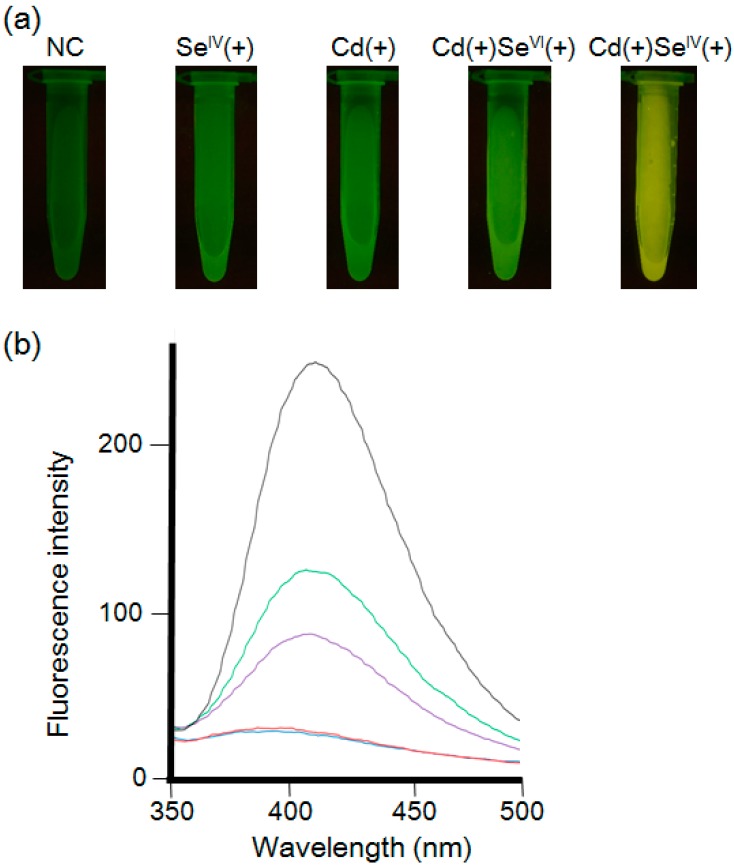
(**a**) *F. oxysporum* after the treatment of cadmium and/or selenium solution observed under the ultra violet (UV) light (wavelength: 365 nm). NC: Negative control without treatment of cadmium and selenium solution. Se^IV^ (+): 5 mM Na_2_SeO_3_ treatment. Cd (+): 5 mM CdCl_2_ treatment. Cd (+) Se^VI^ (+): 5 mM CdCl_2_ and 5 mM Na_2_SeO_4_ treatment. Cd (+) Se^IV^ (+): 5 mM CdCl_2_ and 5 mM Na_2_SeO_3_ treatment; (**b**) The fluorescence spectra (excitation at 310 nm) of extracts from the mycelial cells in each condition using ultrasonication. Bold black line: 5 mM Cd (+) Se^IV^ (+). Green line: Cd (+). Purple line: Cd (+) Se^VI^ (+). Red line: Se^IV^ (+). Blue line: negative control.

Initially, the fluorescence intensities were compared when the mycelia were incubated with the following solutions: distilled water (control); 5 mM Na_2_SeO_3_ (Se^IV^ (+)); 5 mM CdCl_2_ (Cd (+)); 5 mM CdCl_2_ and 5 mM Na_2_SeO_4_ (Cd (+) Se^VI^ (+)); 5 mM CdCl_2_ and 5 mM Na_2_SeO_3_ (Cd (+) Se^IV^ (+)) (Figure 2). Observations with the transilluminator showed that the control exhibited no fluorescence, indicating there were no appreciable endogenous fluorescent substances in the mycelial cell. Similar results were observed for mycelia cells treated with Se^IV^ (+). Treatments with Cd (+), Cd (+) Se^IV^ (+) and Cd (+) Se^VI^ (+) resulted in the fluorescence of mycelial cells.

**Figure 3:**
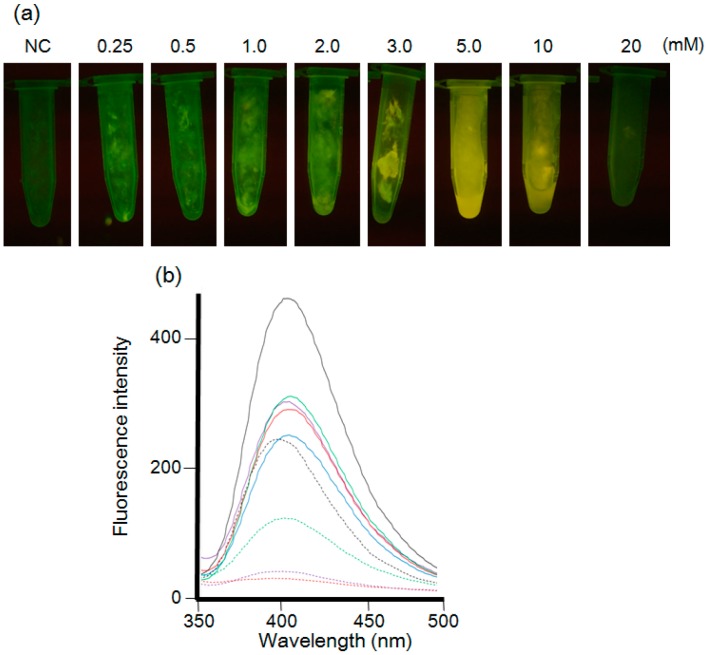
(**a**) *F. oxysporum* after the treatment of various concentrations of CdCl_2_ and Na_2_SeO_3_ solutions observed under the UV light (wavelength: 365 nm). The concentration ratio between CdCl_2_ and Na_2_SeO_3_ kept 1:1. NC: Negative control without treatment of cadmium and selenium solution. From the left side, each tube indicated 0.25, 0.5, 1, 2, 3, 5, 10 and 20 mM solutions, respectively; (**b**) The fluorescence spectra (excitation at 310 nm) of extracts from the mycelial cells in each condition using ultrasonication. Black line: 5 mM. Green line: 2 mM. Red line: 0.5 mM. Purple line: 0.25 mM. Blue line: 1 mM. Black broken line: 3 mM. Green broken line: 10 mM. Purple broken line: 20 mM. Red broken line: negative control.

The fluorescence spectra (excitation wavelength of 310 nm) of the mycelial cell extracts prepared by ultrasonic homogenation are shown in Figure 2b. The spectra with the maximum fluorescence at 410 nm showed that Cd (+) Se^IV^ (+) treatment resulted in the highest fluorescence intensity. These results suggested that Na_2_SeO_3_ is more efficient in producing fluorescence in mycelial cells than was Na_2_SeO_4_. To make the CdSe quantum dots, *F. oxysporum* reduced the Se(IV) or Se(VI) to Se^2−^ using its metabolites. We optimized to use Se(IV), because it is easier to reduce Se(IV) to Se^2−^ than Se(VI). Next, the concentration of cadmium and selenium (IV) ions was optimized. The mycelial cells were suspended in each solution containing an equimolar concentration (0.25, 0.5, 1, 2, 3, 5, 10, or 20 mM) of Cd (+) and Se^IV^ (+) (Figure 3). In the concentration of the 0.25, 0.5, 1, 2, 3 and 10 mM, the mycelial cells of *F. oxysporum* were accumulated in the culture medium. The fluorescence intensity in the transilluminator and spectra of the mycelial cell increased in a dose-dependent manner and maximized at 5 mM Cd (+) Se^IV^ (+) (Figure 3b). The decreased fluorescence intensity at concentrations higher than 10 mM suggested that a high concentration of cadmium and selenium ions might suppress the metabolism of *F. oxysporum* and inhibit fluorescence.

**Figure 4:**
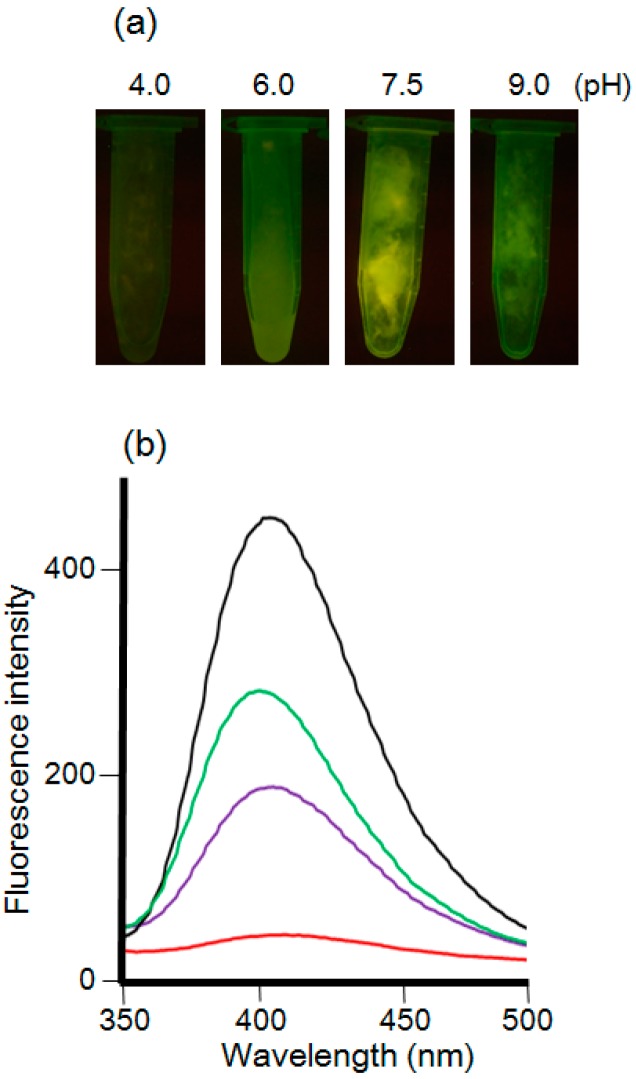
(**a**) *F. oxysporum* after the treatment of 5 mM CdCl_2_ and 5 mM Na_2_SeO_3_ in various conditions of pH observed under the UV light (wavelength: 365 nm). From the left side, each tube indicated pH 4.0, pH 6.0, pH 7.5 and pH 9.0 solutions, respectively; (**b**) The fluorescence spectra (excitation at 310 nm) of extracts from the mycelial cells in each condition using ultrasonication. Black line: pH 7.5. Green line: pH 9. Purple line: pH 6. Red line: pH 4.

The pH of the solution was also optimized. The mycelial cells were suspended in each of the solutions of pH 4.0, 6.0, 7.5, and 9.0, which were prepared using 50 mM potassium hydrogen phthalate, 50 mM sodium phosphate, 50 mM HEPES, and 50 mM Ches, respectively (Figure 4). The transilluminator and fluorescence spectra showed that pH 7.5 resulted in the highest fluorescence intensity.

**Figure 5:**
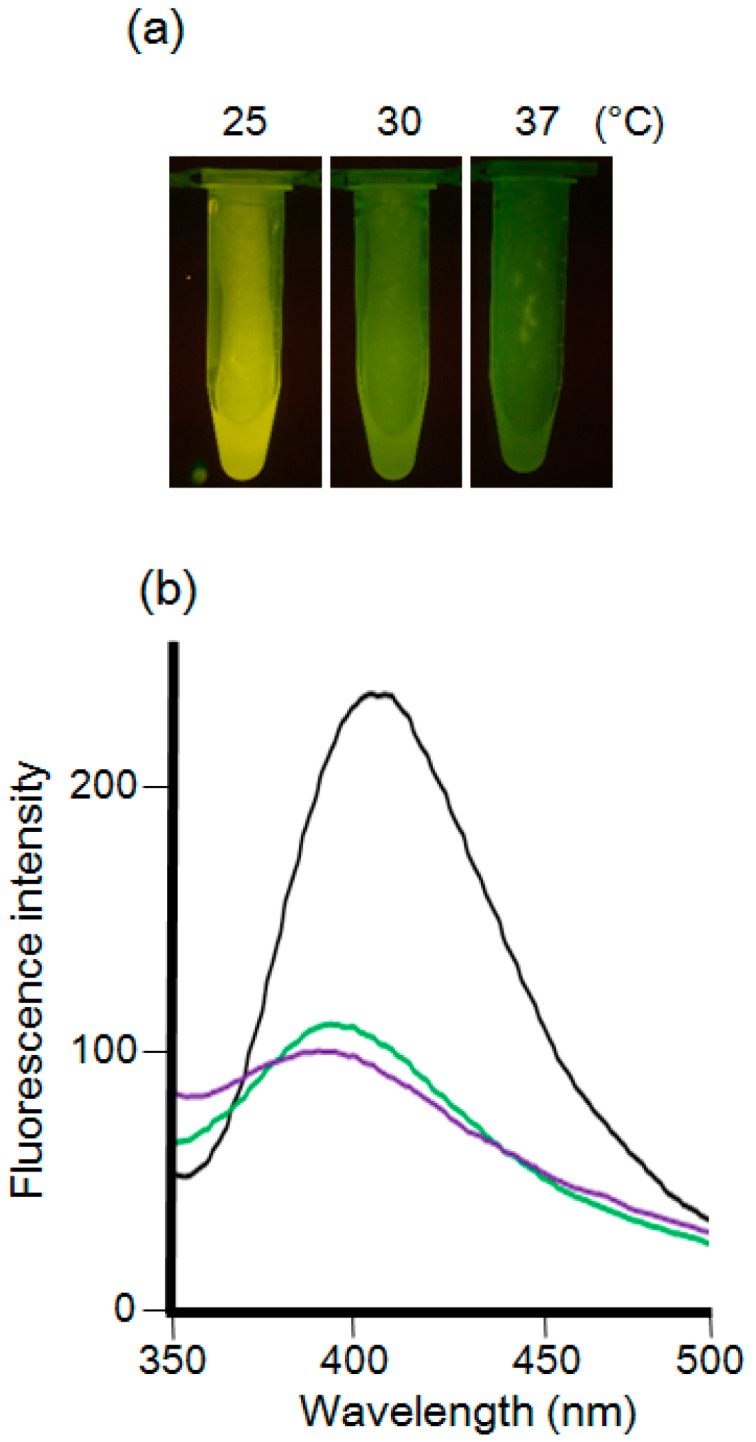
(**a**) *F. oxysporum* after the treatment of 5 mM CdCl_2_ and Na_2_SeO_3_ (pH 7.5) at various temperatures observed under the UV light (wavelength: 365 nm). From the left side, each tube indicated 25, 30 and 37 °C, respectively; (**b**) The fluorescence spectra (excitation at 310 nm) of extracts from the mycelial cells in each condition using ultrasonication. Black line: 25 °C. Green line: 30 °C. Purple line: 37 °C.

Finally, the incubation temperature was optimized. Mycelial cells suspended in a 5 mM Cd (+) Se^IV^ (+) solution (pH 7.5) were incubated at 25, 30, or 37 °C (Figure 5). The transilluminator and fluorescence spectra showed that, when incubated at 30 and 37 °C, the fluorescence intensities of mycelial cells were much lower than when incubated at 25 °C. The incubation at 30 and 37 °C shifted the maximum fluorescence wavelength to 390 nm suggesting that smaller sized CdSe quantum dots were synthesized. The high temperature increased the speed of CdSe precipitation in the solution. The high speed of precipitation might induce the formation of smaller particles, or the different incubation temperatures may change the metabolism of *F. oxysporum*. Given these results, we decided to use the 5 mM Cd (+) Se^IV^ (+) solution (pH 7.5) at 25 °C for the production of fluorescence in *F. oxysporum* mycelial cells.

From these results, the fluorescence intensity depends on the viability of *F. oxysporum*. High concentration of Cd and Se ions, high temperature and low and high pH inhibited the metabolism of *F. oxysporum* and decreased the amounts of synthesized CdSe quantum dots. On the other hand, low concentration of Cd and Se did not supply enough materials to synthesize the big amounts of CdSe quantum dots.

**Figure 6:**
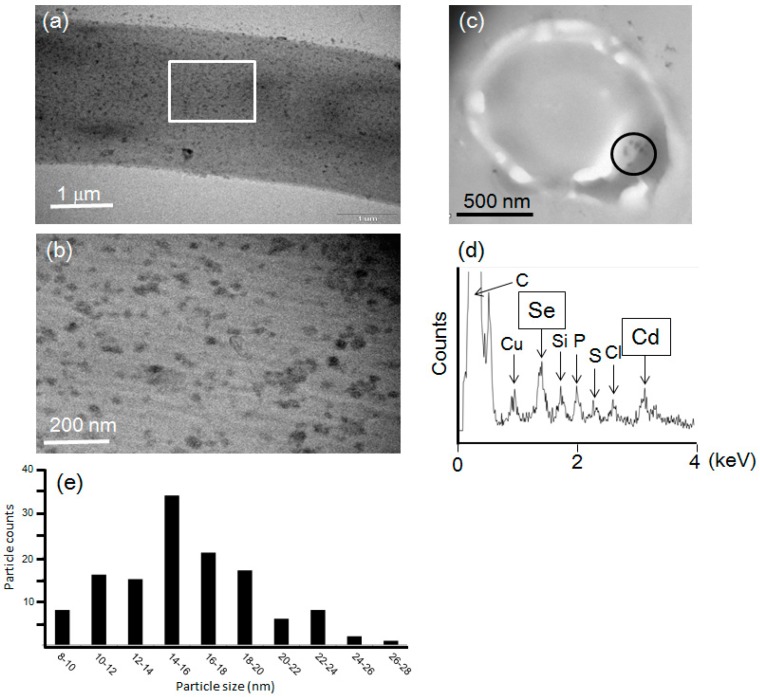
(**a**) Transmission Electron Microscopy (TEM) observation of the mycelial cells of *F. oxysporum* after treatment of a 5 mM Cd (+) Se^IV^ (+) solution (pH 7.5) at 25 °C. The white rectangle region was magnified in (**b**); (**b**) The magnification image of the white rectangle region in (**a**); (**c**) The ultra-thin cross-section of the mycelial cells of *F. oxysporum* after treatment of a 5 mM Cd (+) Se^IV^ (+) solution (pH 7.5) at 25 °C observed by TEM. The black circled region was analyzed by energy-dispersive spectrometer (EDS); (**d**) The EDS spectrum from the black circled region in (**c**); (**e**) The size distribution of nanoparticles in the image of (**b**).

To reveal the fluorescent substances in *F. oxysporum*, the mycelial cells were observed by TEM (Figure 6). Dried mycelial cells incubated with a 5 mM Cd (+) Se^IV^ (+) solution (pH 7.5) at 25 °C were applied to the TEM grid. TEM observation indicated numerous black nanoparticles (Figure 6a,b). Because it is difficult to determine the localization of black nanoparticles in mycelial cells, we prepared ultra-thin cross-sections of the fixed mycelial cells in epoxy resin with an ultra-microtome. Using these cross-sections, black nanoparticles were observed inside the mycelial cells (Figure 6c). An EDS analysis showed that the black nanoparticles were mainly composed of cadmium and selenium, indicating that they were CdSe quantum dots (Figure 6d). Other elements (C, Cu, Si, P, S and Cl) except for cadmium and selenium were detected. Since the black particles were synthesized in the cytoplasm of the cell, C, P, S and Cl, derived from the organic components in the cell, were detected. On the other hand, Cu and Si were contaminants from the grid or resin. These results suggested that CdSe quantum dots were synthesized in the cytoplasm after the uptake of cadmium and selenium ions into the cell. The distribution of particle size in the TEM image was analyzed using ImageJ (Figure 6e). The smallest particle size was about 8 nm. The 14–16 nm particle size was a major distribution. Since some particles aggregated each other, such particles were excluded for the counts.

**Figure 7:**
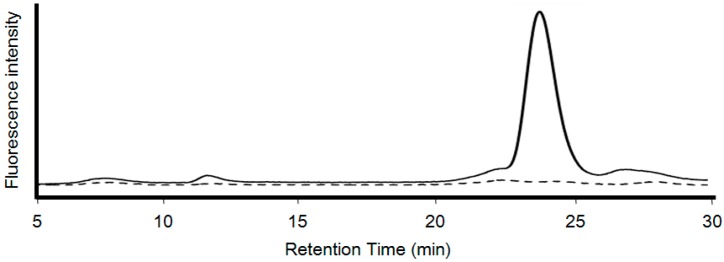
The extracts from *F. oxysporum* after ultrasonication were subjected to gel filtration chromatography using a TSKgel G2000SWXL (7.8 mm × 300 mm, TOSOH). Elution was performed with a 50 mM ammonium acetate buffer solution (pH 5.7) at a flow rate of 0.6 mL/min and monitored by measuring fluorescence at excitation 310 nm/emission 410 nm. Black line: 5 mM Cd (+) Se^IV^ (+) solution (pH 7.5) at 25 °C treatment. Black broken line: Negative control without treatment of cadmium and selenium solution.

To purify the CdSe quantum dots, the extracts of the mycelial cells, following ultrasonication, were subjected to gel-filtration column chromatography (Figure 7). The fluorescence intensity was monitored at 410 nm with an excitation wavelength of 310 nm. There were no peaks from mycelial cell extracts treated with the HEPES buffer (pH 7.5) without cadmium and selenium ions at 25 °C. A sharp peak with a retention time of 24 min was detected from extracts of the cells treated with a 5 mM Cd (+) Se^IV^ (+) solution (pH 7.5) at 25 °C. These results suggest that the size and shape of the CdSe quantum dots are uniform when synthesized by *F. oxysporum*.

**Figure 8:**
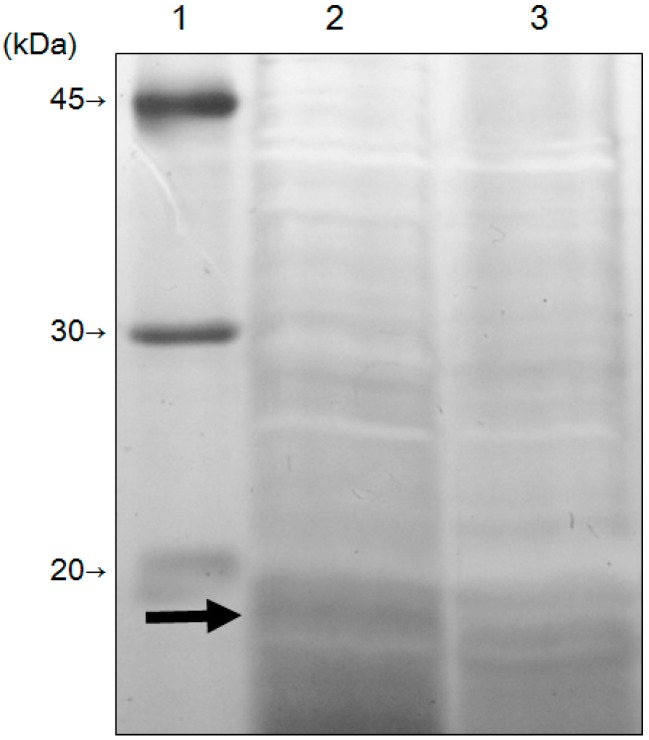
Sodium dodecyl sulfate-polyacrylamide gel electrophoresis (SDS-PAGE) of the extracts from *F. oxysporum* before (lane 2) and after (lane 3) treatment of a 5 mM Cd (+) Se^IV^ (+) solution (pH 7.5) at 25 °C. Lane 1 showed the marker. Black arrow indicated that the specific band in the extracts from *F. oxysporum* before treatment of a 5 mM Cd (+) Se^IV^ (+) solution (pH 7.5) at 25 °C.

**Figure 9:**
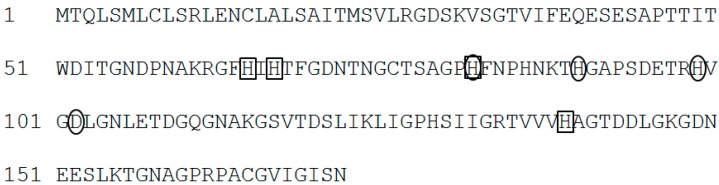
The amino acid sequence of SOD identified from *F. oxysporum* (FoSOD). Black rectangles showed the Cu-binding residues. Black circles showed the Zn-binding residues.

To reveal the molecular mechanism of CdSe quantum dots synthesis, the proteins extracted from *F. oxysporum* before and after treatment with 5 mM Cd (+) Se^IV^ (+) solution (pH 7.5) at 25 °C were analyzed by SDS-PAGE (Figure 8). The band around 20 kDa disappeared in the fraction of extracted proteins after treatment with the Cd (+) Se^IV^ (+) solution, indicating that the expression of this 20-kDa protein is related to the synthesis of the CdSe quantum dots. An LC/MS/MS analysis of this 20-kDa band revealed it to be superoxide dismutase (SOD) (Table 1). The detected peptide fragments covered 54.7% of the region of the amino acid sequence. The score that showed the probability of identification was 31.8%. SOD catalyzes the disproportionation of the superoxide radical, O_2_^−^, yielding dioxygen and hydrogen peroxide (2O_2_^−^ + 2H^+^ → O_2_ + H_2_O_2_) [19]. Eukaryotes have a manganese-containing form of SOD in the mitochondria and a copper- and zinc-containing form in the cytoplasm [20]. SOD identified from *F. oxysporum* (FoSOD) has a conserved sequence that binds to copper and zinc, indicating that FoSOD acts as a Cu–Zn SOD in the cytoplasm (Figure 9). Treatment with the Cd (+) Se^IV^ (+) solution reduced the amount of FoSOD protein.

**Figure 10:**
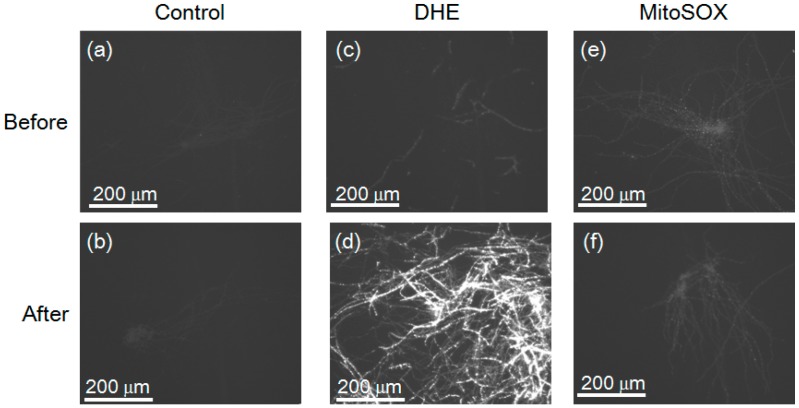
Superoxide levels in the mycelial cells of *F. oxysporum* before and after treatment of a 5 mM Cd (+) Se^IV^ (+) solution (pH 7.5) at 25 °C were observed by the fluorescence microscope. (**a**) The mycelial cells of *F. oxysporum* incubated without any reagents before treatment of a cadmium and selenium solution; (**b**) The mycelial cells of *F. oxysporum* incubated without any reagents after treatment of a cadmium and selenium solution; (**c**) The mycelial cells of *F. oxysporum* incubated with dihydroethidium (DHE) before treatment of a cadmium and selenium solution; (**d**) The mycelial cells of *F. oxysporum* incubated with DHE after treatment of a cadmium and selenium solution; (**e**) The mycelial cells of *F. oxysporum* incubated with mitoSOX before treatment of a cadmium and selenium solution; (**f**) The mycelial cells of *F. oxysporum* incubated with mitoSOX after treatment of a cadmium and selenium solution.

The washed mycelial cells of *F. oxysporum* before and after treatment with the Cd (+) Se^IV^ (+) solution were mixed with mitoSOX and dihydroethidium (DHE) to detect cellular superoxide (Figure 10). The mitoSOX identified superoxide in the mitochondria, whereas DHE identified superoxide in the cytoplasm [21,22]. We set the same exposure time for the pictures to compare the intensity of fluorescence. Washed mycelial cells of *F. oxysporum* before and after treatment with the Cd (+) Se^IV^ (+) solution without mitoSOX and DHE showed no fluorescence (Figure 10a,b), suggesting that the mycelial cells do not contain appreciable amounts of endogenous fluorescent substances. The washed mycelial cells of *F. oxysporum* following treatment with the Cd (+) Se^IV^ (+) solution exhibited the strongest fluorescence with a DHE reagent (Figure 10d). On the other hand, weak fluorescence was detected before the treatment (Figure 10c). In the mitoSOX experiment (Figure 10e,f), the weak fluorescence was also detected suggesting that superoxide did not occur in the mitochondria. These results suggest that, following treatment with the Cd (+) Se^IV^ (+) solution, *F. oxysporum* accumulated superoxide in the cytoplasm. This result is consistent with the decrease in cytoplasmic FoSOD of *F. oxysporum* after treatment with the Cd (+) Se^IV^ (+) solution. Recently, some previous reports suggested that superoxide is related to a reduction of metal ions. Rose et al. (2005) showed that superoxide is used for the reduction of Fe(III) in *Lyngbya majuscule* to absorb the Fe(II) using a Fe(II) transporter [23]. Hansel et al. (2012) reported that *Stilbella aciculosa* oxidizes Mn(II) to Mn oxides by producing extracellular superoxide during cell differentiation [24]. Yin et al. (2016) revealed that superoxide reduced Ag^+^ ion to make the silver nanoparticles in *F. oxysporum* [25]. Superoxide might be related to the reduction of Se(IV). Increased cytoplasmic superoxide may induce the expression of various reductases and secondary metabolites, which may play a role in the synthesis of CdSe quantum dots in *F. oxysporum*. To reveal the detailed molecular mechanism, however, further analyses will be needed.

## 4. Conclusions

In this study, we succeeded in synthesizing CdSe quantum dots using *F. oxysporum*. An investigation of various culture conditions revealed that a 5 mM Cd (+) Se^IV^ (+) solution (pH 7.5) at 25 °C was the optimal condition for CdSe synthesis. TEM observations showed that the size of the CdSe quantum dots was less than 20 nm, and these dots were purified using a gel filtration column. To further assess optimal conditions for the synthesis of CdSe quantum dots, the components of *F. oxysporum* mycelial cells before and after treatment with the Cd (+) Se^IV^ (+) solution were analyzed by SDS-PAGE. Cu–Zn SOD in the cytoplasm decreased following Cd (+) Se^IV^ (+) treatment. At the same time, the level of superoxide increased in the cytoplasm, which may be related to the formation of CdSe quantum dots in *F. oxysporum*.

## Figures and Tables

**Table 1:** Result of liquid chromatography/mass/mass (LC/MS/MS) analysis of superoxide dismutase.

Accession	Obtained Peptides
FOXG_03076P0	VSGTVIFEQESESAPTTITWDITGNDPNAKR
	HVGDLGNLETDGQGNAKGSVTDSLIK
	HVGDLGNLETDGQGNAK
	LIGPHSIIGR
	THGAPSDETR
	TGNAGPRPAcGVIGISN
	GSVTDSLIK
	VSGTVIFEQESESAPTTITWDITGNDPNAK
